# Navigating the shadows: medical professionals’ values and perspectives on end-of-life care within pediatric intensive care units in Croatia

**DOI:** 10.3389/fped.2024.1394071

**Published:** 2024-08-12

**Authors:** Marko Curkovic, Filip Rubic, Ana Jozepovic, Milivoj Novak, Boris Filipovic-Grcic, Julije Mestrovic, Kristina Lah Tomulic, Branimir Peter, Diana Spoljar, Štefan Grosek, Sunčana Janković, Jurica Vukovic, Mirjana Kujundžić Tiljak, Andrija Štajduhar, Ana Borovecki

**Affiliations:** ^1^ University Psychiatric Hospital Vrapče, Zagreb, Croatia; ^2^ School of Medicine, University of Zagreb, Zagreb, Croatia; ^3^ Department of Pediatrics, University Hospital Centre Zagreb, Zagreb, Croatia; ^4^ Department of Anesthesiology, University Hospital Centre Zagreb, Zagreb, Croatia; ^5^ Department of Pediatrics, University Hospital Centre Split, Split, Croatia; ^6^ Department of Pediatrics, University Hospital Centre Rijeka, Rijeka, Croatia; ^7^ Department of Gynecology and Obstetrics, University Hospital Centre Rijeka, Rijeka, Croatia; ^8^ Palliative Care Service, Community Health Center Zagreb, Zagreb, Croatia; ^9^ Department of Perinatology, Division of Gynaecology and Obstetrics, University Medical Centre Ljubljana, Ljubljana, Slovenia; ^10^ Andrija Štampar School of Public Health, School of Medicine, University of Zagreb, Zagreb, Croatia

**Keywords:** pediatrics, intensive care, intensive care units, end-of-life, ethics, withholding, withdrawing, healthcare professionals

## Abstract

**Background and aim:**

This study explores healthcare professionals’ perspectives on end-of-life care in pediatric intensive care units (ICUs) in Croatia, aiming to illuminate their experiences with such practices, underlying attitudes, and major decision-making considerations. Amid the high variability, complexity, and emotional intensity of pediatric end-of-life decisions and practices, understanding these perspectives is crucial for improving care and policies.

**Methods:**

The study utilized a cross-sectional survey intended for physicians and nurses across all pediatric ICUs in Croatia. It included healthcare professionals from six neonatal and four pediatric ICUs in total. As the data from neonatal and pediatric ICUs were examined jointly, the term pediatric ICU was used to denominate both types of ICUs. A statistical analysis was performed using Python and JASP, focusing on professional roles, professional experience, and regional differences.

**Results:**

The study included a total of 103 participants (with an overall response rate—in relation to the whole target population—of 48% for physicians and 29% for nurses). The survey revealed diverse attitudes toward and experiences with various aspects of end-of-life care, with a significant portion of healthcare professionals indicating infrequent involvement in life-sustaining treatment (LST) limitation discussions and decisions, as well as somewhat ambiguous attitudes regarding such practices. Notably, discrepancies emerged between different professional roles and, in particular, regions, underscoring the high variability of LST limitation-related procedures.

**Conclusions:**

The findings highlight a pressing need for more straightforward guidelines, legal frameworks, support mechanisms, and communication strategies to navigate the complex terrain of rather burdensome end-of-life pediatric care, which is intrinsically loaded with profound ethical quandaries.

## 1 Introduction

In Western, high-income countries, most child and adolescent (pediatric) deaths take place within hospitals or, more precisely, intensive care units (ICUs) (1, 2). A significant part, if not the majority, of those deaths, occurs after withholding, non-escalation, or withdrawal of life-sustaining treatments (LST) (3–6). LST implies all medical interventions or procedures used to prolong life, that is, delay death regardless of the potential influence on underlying causes, which may or may not be present (7).

Therefore, despite advances in medical technologies and treatments that have improved overall survival rates and quality of life, a considerable subset of cases remain where available interventions are futile, shifting the focus from cure toward comfort and palliation (8, 9). The proper management of end-of-life issues is thus a critical component of (intensive) pediatric care.

In the emotionally charged environment of pediatric ICUs, healthcare professionals face multiple challenges as, while providing cure and care, they must also navigate the complexities of end-of-life decision-making (10, 11). Their attitudes and perspectives, influenced by factors such as demographics, professional roles, and social and cultural context, significantly impact the care provided (12–15). Given the importance, complexity, and substantial variability of end-of-life care in pediatric ICUs, understanding these attitudes and perspectives is essential for developing and adopting support mechanisms, guidelines, and policies aimed at optimizing end-of-life care.

End-of-life decision-making in Croatia may differ due to specific local factors, including those related to social, cultural, and healthcare systems. Croatia is the youngest member of the European Union (EU). However, despite being part of the EU since 2013, Croatia still inherits some features of the former socialist system it was part of, for example, corruption and mistrust in institutions (16). Croatia has a relatively homogenous population. According to the last census in 2021, the share of Croats in Croatia is 91.63% (17). Regarding religion, Christians make up 87.39% of its population 78.97 % of them being Catholics (17). The average age of the population is 44.3 years, which places Croatia among the oldest nations in Europe (while causes of aging are both negative natural increase rates and pronounced emigration (18).

Croatia has a public-private mix healthcare system, but still, most of the care for most of the population is provided within the public system, especially its clearly non-profitable segments, while more profitable ones are under pressure of commodification (19). Nominally, appropriate healthcare within the public system is accessible in Croatia, but there are marked differences in accessibility between rural and urban areas (20).

In Croatia, any kind of medically assisted dying, that is, euthanasia and assisted suicide, is forbidden by Criminal law. However, legal provisions regarding other segments of end-of-life care are rather fragmented. For example, anticipatory decision-making, like advance care directives (ACD), is *de iure* envisaged in the Family law but is *de facto* not implementable due to the lack of necessary additional legal provisions (21). In addition, by relevant laws, patients are granted the right to accept or refuse any given medical procedure, but only if it does not threaten their life and health. Put simply, despite their daily presence in a clinical context, the practices of LST limitation are not regulated by law in Croatia. Nonetheless, palliative care is considered an essential part of healthcare.

So far, several studies have dealt with end-of-life issues in Croatia, employing qualitative and quantitative methodologies. Research on public attitudes, without delineation between adult and pediatric applications, showed a rather low level of acceptance of LST limitation as well as euthanasia and assisted suicide (21). On the other hand, most Croatian ICU professionals consider the limitation of LST as ethically acceptable, although such practices are seemingly less often encountered than in most other countries (22). However, almost all previous studies found a high variability of both end-of-life attitudes and practices (22–24).

This study is part of a research project founded by the Croatian Science Foundation entitled “Values and Decisions at the End of Life” (VAL-DE-END), which aims to comprehensively investigate the values, attitudes, and experiences related to end-of-life practices in ICUs across Croatia.

This article reports findings from a cross-sectional survey of healthcare professionals working in pediatric ICUs in Croatia. It complements previous qualitative research and seeks to provide further insights into the particularities of end-of-life care in pediatric ICUs in this context (24). To the best of our knowledge, this is the first study of its kind in Croatia.

In line with the above, the main aim of this study is to explore further the delicate nuances of healthcare professionals’ experiences, challenges faced, and the strategies employed to manage these demanding yet critical aspects of pediatric care.

## 2 Methods

A cross-sectional study using a questionnaire was conducted. The target population was healthcare professionals (physicians and nurses) working in Croatia's pediatric ICUs (PICUs), encompassing both neonatal (NICUs) and PICUs. Participants were recruited from all such ICUs, excluding those from University Clinical Hospital Osijek as a result of the lack of ethical committee approval.

At the time of the study, Croatia had seven NICUs and five PICUs, all operating at the tertiary level within clinical hospitals (24). This comprised 72 physicians and 325 nurses as the overall target population. With the exclusion of Osijek, the eligible population was 64 physicians and 283 nurses.

This study used equal methodology in terms of questionnaire distribution and collection, as one was conducted among healthcare professionals working in adult ICUs in Croatia [and is reported in more detail in the study by Špoljar et al. (22)]. In short, questionnaires were distributed through ICU directors following thorough instructions from researchers. The directors locally coordinated distribution and collection, ensuring anonymity and voluntary participation. This process occurred between December 2018 and December 2019.

The questionnaire used was similar to one previously employed in a study on adult ICU healthcare professionals within the same research project. The questionnaire was only slightly adjusted, mainly in the introductory part concerning general data, for the pediatric ICU context to allow comparison between pediatric and adult ICU professionals.

The questionnaire, as previously discussed in more detail (22), was consensually coopted from its initial developers (25, 26), who previously used it in a nationwide sample of healthcare professionals working in adult ICU and pediatric ICU in Slovenia. The rationale for using that specific questionnaire relies on the fact that Slovenia and Croatia are neighboring countries that share a significant part of recent historical and current sociocultural context, thus allowing for a more straightforward comparison. Before applying to this research project, the questionnaire was doubly translated and back-translated, further adjusted, pilot-tested, and finally validated (22).

The translated survey questionnaire used in this study is available in the Supplementary Material (Supplementary Data Sheet 1, Survey Questionnaire).

## 3 Analysis

The statistical analysis was conducted using the Python programming language and JASP software (version 0.18.3). A comprehensive descriptive analysis was performed on the entire dataset. The primary dependent variables analyzed included professional role/status (dichotomized as physicians or nurses), years of professional experience, and geographic location (categorized as Zagreb, Rijeka, or Split). Although other variables were present in the questionnaire, they were excluded from the analysis due to either significant correlation with the primary variables (e.g., age and ICU-specific experience with overall professional tenure) or insufficient representation within certain categories (such as the male gender demographic). This decision was informed by both the research group's prior studies and established literature, indicating these selected variables as critical determinants. Continuous variables were described using statistical measures such as mean, median, standard deviation, and interquartile range where appropriate. Correlation analyses employed Pearson's r and Spearman's rho coefficients. Categorical variables were summarized by frequency counts (*n*) and percentages. Differences between two groups were assessed using the Mann–Whitney *U*-test, while the ANOVA and Kruskal–Wallis tests were applied for comparisons across more than two groups, incorporating Tukey's *post hoc* correction for multiple comparisons. A significance threshold was established at *p* ≤0.05.

## 4 Results

### 4.1 Characteristics of study participants

The study was conducted in six NICUs and four PICUs in five different hospitals (three from Zagreb and one each from Rijeka and Split). The response rate was 48% for physicians (31 out of 64 eligible) and 29% for nurses (82 out of 283 eligible), totaling 103 participants. The majority were female (92%), with 27.4% being physicians. Among the nurses, 32.7% had college degrees, 28.3% were high school graduates, and 11.5% held university degrees. The mean age of participants was 38.9 years (SD ±11.7), with a mean of 16.8 years of working experience (SD ±12.2). Most participants (*n* = 85, 8%) worked in the ICU regularly, with the largest group from Zagreb (58.4%).

All the other characteristics of study participants are presented in Table 1.

**Table 1:** Characteristics of study participants.

		Frequency	Percent
Hospital	UCH Holy Spirit	27	23.89
	UCH Zagreb	32	28.31
	UCH Sisters of Mercy	7	6.19
	UCH Split	25	22.12
	UCH Rijeka	22	19.46
Region	Zagreb	66	58.40
	Split	25	22.12
	Rijeka	22	19.46
Gender	Female	104	92.03
	Male	9	7.96
Vocation	Nurse	82	72.56
	Nurse-college graduate	37	32.74
	Nurse-high school graduate	32	28.31
	Nurse-university graduate	13	11.50
	Physician	31	27.43
Type of work in ICU	Occasional	16	14.15
	Regular	97	85.84
Years of practice (as nurse of physician)	Median	15	
	Standard deviation	12.23	
	Mean	16.81	
Age	Median	38	
	Standard deviation	11.70	
	Mean	38.99	

### 4.2 Experiences with LST limitation at designated ICUs

Approximately half of healthcare professionals reported that they do not attempt cardiopulmonary resuscitation (DNACPR) and that withholding LST decisions were made in their ICUs, although such decisions were fairly rare (41.6% and 42.5%, respectively). Over one-third indicated such decisions were never made.

The majority of healthcare professionals stated that decisions to withdraw artificial ventilation (58.4%), antibiotics (57.5%), endotracheal tube (71.7%), and hydration (83.2%) are never made. However, decisions to withdraw inotropes were more commonly encountered, albeit on rare occasions (37.2%).

Physicians reported more often experiences with withholding LST (*U* = 840.5; *p* < 0.001), withdrawing of artificial ventilation (*U* = 840.5; *p* = 0.066), and withdrawing inotropes (*U* = 712; *p* = 0.008).

A significant majority of healthcare professionals, three-quarters of them, reported never being involved in the LST limitation decision-making process, with notable differences between physicians (51.6% involvement) and nurses (9.8% involvement) (*U* = 629.5; *p* < 0.001).

Physicians were generally more often seen as the primary initiators of LST discussions (60.2%), and this was also more commonly reported by physicians themselves (*U* = 864; *p* = 0.038). LST limitation decision-making process predominantly involved physicians (73.4%), with a much lesser involvement of nurses (29.2%) and ethics committees (15%). Family members and/or legal guardians were frequently included in those decision-making processes (82.3% usually or always included).

In general, a significant minority (82.3%) never consulted ethics committees. Physicians reached out to ethics committees more often than nurses (29% vs. 13.4%), although this was not statistically significant. Knowledge about whom to contact for ethical dilemmas was limited, with only one-third of healthcare professionals knowing the appropriate contacts.

Compliance with DNACPR decisions is relatively high (63.7% always complied), while, expectedly, personal disagreement with LST limitation decisions was relatively low (44.2% never disagreed, 47.8% rarely disagreed).

Lack of consensus was rare among both physicians and family members (69.9%) and even more so among physicians themselves (77.9%). Physicians, however, more frequently reported a lack of consensus between physicians and family members/legal guardians (*U* = 849; *p* = 0.004).

Decisions were either not recorded or documented equally for DNACPR (38.1%) and LST limitations (35.4%). Nonetheless, it was implemented quickly once a decision was made (66.3%).

For more details on participants’ answers, refer to Table 2.

**Table 2:** Experiences of healthcare professionals with LST limitation at designated ICUs: description and analysis according to vocation (nurses/physicians).

Question	Answer	All, *N* (%)	Physician, *N* (%)	Nurse, *N* (%)	Mann–Whitney	
					*U*	*p*
I.11. Knowledge on whom to reach when encountering ethical dilemmas	0^a^	1 (0.9%)	1 (3.2%)	0 (0.0%)		* *
	No	73 (64.6%)	18 (58.1%)	55 (67.1%)		
	Yes	39 (34.5%)	12 (38.7%)	27 (32.9%)		
I.12. Ever contacted ethical committee	No	93 (82.3%)	22 (71.0%)	71 (86.6%)		
	Yes	20 (17.7%)	9 (29.0%)	11 (13.4%)		
II.1.1. Experiencing DNACPR decisions	0^a^	10 (8.8%)	3 (9.7%)	7 (8.5%)	923.000	0.302
	Never	44 (38.9%)	9 (29.0%)	35 (42.7%)		
	Rarely	47 (41.6%)	16 (51.6%)	31 (37.8%)		
	Frequently	12 (10.6%)	3 (9.7%)	9 (11.0%)		
II.1.2. Experiencing withholding LST	0^a^	14 (12.4%)	3 (9.7%)	11 (13.4%)	606.500	<.001
	Never	41 (36.3%)	3 (9.7%)	38 (46.3%)		
	Rarely	48 (42.5%)	22 (71.0%)	26 (31.7%)		
	Frequently	10 (8.8%)	3 (9.7%)	7 (8.5%)		
II.1.3. Experiencing withdrawing of artificial ventilation	0^a^	10 (8.8%)	3 (9.7%)	7 (8.5%)	840.500	0.066
	Never	66 (58.4%)	14 (45.2%)	52 (63.4%)		
	Rarely	30 (26.5%)	11 (35.5%)	19 (23.2%)		
	Frequently	7 (6.2%)	3 (9.7%)	4 (4.9%)		
II.1.4. Experiencing withdrawing of endotracheal tube	0^a^	10 (8.8%)	3 (9.7%)	7 (8.5%)	985.500	0.506
	Never	81 (71.7%)	21 (67.7%)	60 (73.2%)		
	Rarely	16 (14.2%)	4 (12.9%)	12 (14.6%)		
	Frequently	6 (5.3%)	3 (9.7%)	3 (3.7%)		
II.1.5. Experiencing withdrawing of inotropes	0^a^	11 (9.7%)	3 (9.7%)	8 (9.8%)	712.000	0.008
	Never	48 (42.5%)	7 (22.6%)	41 (50.0%)		
	Rarely	42 (37.2%)	16 (51.6%)	26 (31.7%)		
	Frequently	12 (10.6%)	5 (16.1%)	7 (8.5%)		
II.1.6. Experiencing withdrawing of antibiotics	0^a^	10 (8.8%)	3 (9.7%)	7 (8.5%)	926.500	0.284
	Never	65 (57.5%)	15 (48.4%)	50 (61.0%)		
	Rarely	30 (26.5%)	11 (35.5%)	19 (23.2%)		
	Frequently	8 (7.1%)	2 (6.5%)	6 (7.3%)		
II.1.7. Experiencing withdrawing of hydration	0^a^	10 (8.8%)	3 (9.7%)	7 (8.5%)	1,023.000	0.688
	Never	94 (83.2%)	25 (80.6%)	69 (84.1%)		
	Rarely	8 (7.1%)	3 (9.7%)	5 (6.1%)		
	Frequently	1 (0.9%)	0 (0.0%)	1 (1.2%)		
II.2. Recording of DNACPR decisions	0^a^	1 (0.9%)	1 (3.2%)	0 (0.0%)	1,230.000	1.000
	No	43 (38.1%)	11 (35.5%)	32 (39.0%)		
	In writing	43 (38.1%)	11 (35.5%)	32 (39.0%)		
	Orally	26 (23.0%)	8 (25.8%)	18 (22.0%)		
II.3. Compliance with DNACPR decisions	0^a^	4 (3.5%)	1 (3.2%)	3 (3.7%)	1,262.000	0.535
	Never	16 (14.2%)	4 (12.9%)	12 (14.6%)		
	Rarely	21 (18.6%)	8 (25.8%)	13 (15.9%)		
	Always	72 (63.7%)	18 (58.1%)	54 (65.9%)		
II.4. Recording of LST limitation decisions	0^a^	1 (0.9%)	0 (0.0%)	1 (1.2%)	1,059.500	0.177
	No instructions used	40 (35.4%)	9 (29.0%)	31 (37.8%)		
	Oral instructions	32 (28.3%)	12 (38.7%)	20 (24.4%)		
	Written instructions	40 (35.4%)	10 (32.3%)	30 (36.6%)		
II.5.1. Involvement in LST limitation decision-making	0^a^	5 (4.4%)	2 (6.5%)	3 (3.7%)	629.500	<.001
	No	84 (74.3%)	13 (41.9%)	71 (86.6%)		
	Yes	24 (21.2%)	16 (51.6%)	8 (9.8%)		
II.6. Initiator of LST limitation discussions	0^a^	29 (25.7%)	7 (22.6%)	22 (26.8%)	864.000	0.038
	Doctor + family/legal guardian	4 (3.5%)	1 (3.2%)	3 (3.7%)		
	Doctor + nurse	2 (1.8%)	0 (0.0%)	2 (2.4%)		
	Doctor's initiative	68 (60.2%)	23 (74.2%)	45 (54.9%)		
	Family/legal guardian's initiative	10 (8.8%)	0 (0.0%)	10 (12.2%)		
II.7.2. LST limitation decision-making process includes physicians (ICU and others involved in treatment)	0^a^	8 (7.1%)	0 (0.0%)	8 (9.8%)	1,307.500	0.229
	Strongly disagree	13 (11.5%)	5 (16.1%)	8 (9.8%)		
	Disagree	5 (4.4%)	1 (3.2%)	4 (4.9%)		
	Undecided	4 (3.5%)	1 (3.2%)	3 (3.7%)		
	Agree	38 (33.6%)	14 (45.2%)	24 (29.3%)		
	Strongly agree	45 (39.8%)	10 (32.3%)	35 (42.7%)		
II.7.3. LST limitation decision-making process includes physicians and nurses	0^a^	15 (13.3%)	3 (9.7%)	12 (14.6%)	861.500	0.341
	Strongly disagree	27 (23.9%)	5 (16.1%)	22 (26.8%)		
	Disagree	18 (15.9%)	5 (16.1%)	13 (15.9%)		
	Undecided	20 (17.7%)	8 (25.8%)	12 (14.6%)		
	Agree	24 (21.2%)	8 (25.8%)	16 (19.5%)		
	Strongly agree	9 (8.0%)	2 (6.5%)	7 (8.5%)		
II.7.4. LST limitation decision-making process includes consultation of ethics committee	0^a^	13 (11.5%)	2 (6.5%)	11 (13.4%)	1,129.500	0.426
	Strongly disagree	43 (38.1%)	13 (41.9%)	30 (36.6%)		
	Disagree	16 (14.2%)	8 (25.8%)	8 (9.8%)		
	Undecided	24 (21.2%)	3 (9.7%)	21 (25.6%)		
	Agree	7 (6.2%)	3 (9.7%)	4 (4.9%)		
	Strongly agree	10 (8.8%)	2 (6.5%)	8 (9.8%)		
II.8. Family members/legal guardians involvement in LST limitation decision-making	0^a^	1 (0.9%)	1 (3.2%)	1 (1.2%)	1,114.000	0.411
	Never	8 (7.1%)	3 (9.7%)	5 (6.1%)		
	Rarely	10 (8.8%)	1 (3.2%)	9 (11.0%)		
	Usually	43 (38.1%)	10 (32.3%)	33 (40.2%)		
	Always	50 (44.2%)	16 (51.6%)	34 (41.5%)		
II.11. Frequency of no consensus among physicians	0^a^	3 (2.7%)	1 (3.2%)	2 (2.4%)	1,348.500	0.154
	Never	11 (9.7%)	5 (16.1%)	6 (7.3%)		
	Rarely	88 (77.9%)	23 (74.2%)	65 (79.3%)		
	Frequently	11 (9.7%)	2 (6.5%)	9 (11.0%)		
II.12. Frequency of no consensus among physicians and family members	0^a^	4 (3.5%)	1 (3.2%)	3 (3.7%)	849.000	0.004
	Never	17 (15.0%)	0 (0.0%)	17 (20.7%)		
	Rarely	79 (69.9%)	24 (77.4%)	55 (67.1%)		
	Frequently	13 (11.5%)	6 (19.4%)	7 (8.5%)		
II.13. Disagreement with LST limitation decisions	0^a^	2 (1.8%)	1 (3.2%)	1 (1.2%)	1,215.500	1.000
	Never	50 (44.2%)	13 (41.9%)	37 (45.1%)		
	Rarely	54 (47.8%)	16 (51.6%)	38 (46.3%)		
	Frequently	7 (6.2%)	1 (3.2%)	6 (7.3%)		
II.15. Time from decision to treatment withdrawal	0^a^	8 (7.1%)	1 (3.2%)	7 (8.5%)		
	Immediately	10 (8.8%)	2 (6.5%)	8 (9.8%)	1,211.000	0.486
	Immediately after decision and family agreement	65 (57.5%)	18 (58.1%)	47 (57.3%)		
	About 6 h	13 (11.5%)	5 (16.1%)	8 (9.8%)		
	About 24 h	12 (10.6%)	4 (12.9%)	8 (9.8%)		
	More than 24 h	5 (4.4%)	1 (3.2%)	4 (4.9%)		

^a^ 0 = Non-response to a given questionnaire item.

### 4.3 Attitudes and values regarding LST limitation

There was significant polarization in attitudes toward the ethical acceptability of LST limitation. Nearly half found it acceptable, while the other half had a completely opposite view. Physicians tended to find LST limitation more ethically acceptable than nurses, although this was not statistically significant.

DNACPR was viewed more favorably, although one-third found it unacceptable, especially among nurses (34.1%) compared to physicians (22.6%).

Participants were divided on whether there is an ethical difference between withholding and withdrawing of LST. Physicians were less likely to see a difference (16.1%) compared to nurses (41.5%), but again with a lack of statistical significance. Most professionals (83.9%) were undecided about the ethical acceptability of limiting hydration.

Participants were also divided in their evaluation of ethical differences between different kinds of end-of-life cases [namely, cases of brain-dead patients, terminal patients, and patients in a (irreversible) vegetative state]. At the same time, physicians were less prone than nurses to consider these cases as equal (25.8% vs. 41.5%). Prognostic certainty and finality of being brain-dead (as opposed to being terminal or in a vegetative state) were among the most often highlighted reasons for unequal evaluation of those cases. A thin majority of our participants (64.6%) considered LST limitation decision-making in pediatric and adult contexts as ethically equal, with nurses being more likely to see a difference (29.3% vs. 16.1%).

A significant majority (80.5%) believed competent patient verbal or written decisions should be respected, though a notable portion of physicians (22.6%) remained undecided. However, participants reported not being very often informed about the wishes of patients, family members, or legal guardians regarding LST limitation (43.4% rarely or very rarely informed).

Most participants, approximately three-quarters, found ACDs valuable and useful in the LST limitation context, although an almost equal share of them have yet to encounter one. Physicians valued ACDs more than nurses (83.3% vs. 69.5%).

Views on one's legal responsibility in the context of LST limitation were also somewhat divided. Physicians were split between feeling individually responsible (45.2%) and undecided (41.9%), while nurses were more evenly distributed between agreement, disagreement, and being undecided.

A detailed list of participants’ responses is shown in Table 3.

**Table 3:** Attitudes of healthcare professionals regarding LST limitation at designated ICUs: description and analysis according to vocation (nurses/physicians).

Question	Answer	All, *N* (%)	Physician, *N* (%)	Nurse, *N* (%)	Mann–Whitney	
					*U*	*p*
III.4. Ethical acceptability of LST limitation	Undecided	7 (6.2%)	1 (3.2%)	6 (7.3%)	1,113.000	0.255
	No	53 (46.9%)	13 (41.9%)	40 (48.8%)		
	Yes	53 (46.9%)	17 (54.8%)	36 (43.9%)		
III.5. Ethical difference between withholding and withdrawing	Undecided	32 (28.3%)	12 (38.7%)	20 (24.4%)	1,285.000	0.926
	No	39 (34.5%)	5 (16.1%)	34 (41.5%)		
	Yes	42 (37.2%)	14 (45.2%)	28 (34.1%)		
III.6. Ethical acceptability of DNACPR	Undecided	11 (9.7%)	4 (12.9%)	7 (8.5%)	1,211.000	0.661
	No	35 (31.0%)	7 (22.6%)	28 (34.1%)		
	Yes	67 (59.3%)	20 (64.5%)	47 (57.3%)		
III.7. Ethical acceptability of limitation of hydration alongside limitation of LST	Undecided	94 (83.2%)	23 (74.2%)	71 (86.6%)	1,114.500	0.122
	No	14 (12.4%)	6 (19.4%)	8 (9.8%)		
	Yes	5 (4.4%)	2 (6.5%)	3 (3.7%)		
III.8. Ethical equality of different end-of-life cases [brain dead, terminal, and patients in (irreversible) vegetative state]	Undecided	26 (23.0%)	11 (35.5%)	15 (18.3%)	1,417.500	0.315
	No	42 (37.2%)	8 (25.8%)	34 (41.5%)		
	Yes	45 (39.8%)	12 (38.7%)	33 (40.2%)		
III.10. Ethical equality of LST limitation decision-making involving adult and pediatric patients	Undecided	11 (9.7%)	3 (9.7%)	8 (9.8%)	1,119.000	0.248
	No	29 (25.7%)	5 (16.1%)	24 (29.3%)		
	Yes	73 (64.6%)	23 (74.2%)	50 (61.0%)		
III.13. Competent patient's expressed (verbally or written) decisions should be respected	Don't know	20 (17.7%)	7 (22.6%)	13 (15.9%)	1,451.500	0.092
	No	2 (1.8%)	2 (6.5%)	0 (0.0%)		
	Yes	91 (80.5%)	22 (71.0%)	69 (84.1%)		
III.16. Frequency of being informed about patient or family wishes regarding LST limitation	0^a^	2 (1.8%)	0 (0.0%)	2 (2.4%)	1,280.000	0.784
	Undecided	10 (8.8%)	2 (6.5%)	8 (9.8%)		
	Very rarely	7 (6.2%)	2 (6.5%)	5 (6.1%)		
	Rarely	42 (37.2%)	12 (38.7%)	30 (36.6%)		
	Often	39 (34.5%)	13 (41.9%)	26 (31.7%)		
	Very often	13 (11.5%)	2 (6.5%)	11 (13.4%)		
III.17. Usefulness of ACD in LST limitation decision-making	0^a^	3 (2.7%)	0 (0.0%)	3 (3.7%)	1,199.000	0.853
	Undecided	21 (18.6%)	2 (6.5%)	19 (23.2%)		
	Not useful at all	1 (0.9%)	0 (0.0%)	1 (1.2%)		
	Not useful	5 (4.4%)	3 (9.7%)	2 (2.4%)		
	Useful	63 (55.8%)	22 (71.0%)	41 (50.0%)		
	Very useful	20 (17.7%)	4 (12.9%)	16 (19.5%)		
III.18. Frequency of encountering ACD	0^a^	2 (1.8%)	0 (0.0%)	2 (2.4%)	1.787	0.077
	Never	84 (74.3%)	27 (87.1%)	57 (69.5%)		
	Rarely	26 (23.0%)	4 (12.9%)	22 (26.8%)		
	Often	1 (0.9%)	0 (0.0%)	1 (1.2%)		
III.19. Existence of (your) individual legal responsibility regarding LST limitation decisions and their implementation	0^a^	3 (2.7%)	0 (0.0%)	3 (3.7%)	996.500	0.107
	Don't know	34 (30.1%)	13 (41.9%)	21 (25.6%)		
	No	32 (28.3%)	4 (12.9%)	28 (34.1%)		
	Yes	44 (38.9%)	14 (45.2%)	30 (36.6%)		

^a^ 0 = Non-response to a given questionnaire item.

**Figure 1:**
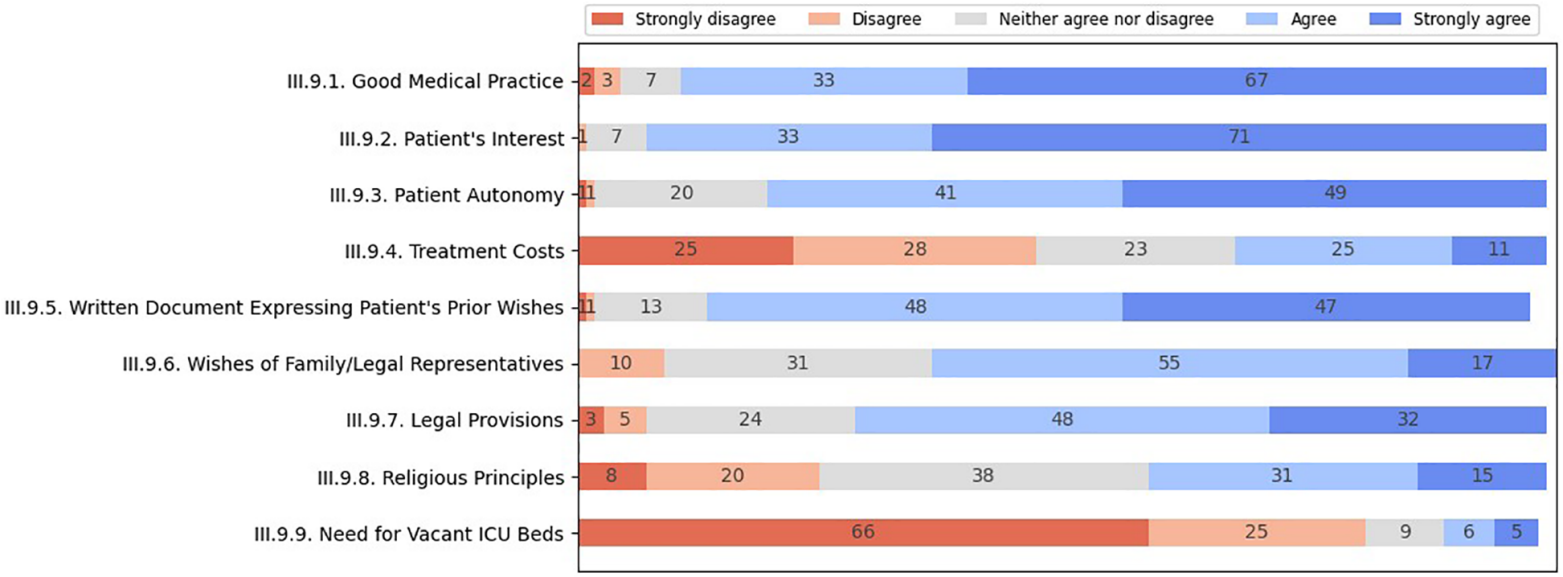
Major considerations of healthcare professionals regarding LST limitation.

When determining the most critical considerations in LST limitation decision-making, healthcare professionals assigned the highest weight (agreeing and strongly agreeing) to the patient's best interest (94%), good medical practice (90%), respect for the patient's autonomy (90%), ACD (95%), legal regulations (80%), and respect for proxy or surrogate wishes (72%) (presented in Figure 1). Somewhat lesser weight was placed on respecting religious principles (46%). However, the religious and cultural beliefs of patients, family members, or legal representatives were considered more important (approximately 60% agreeing and strongly agreeing) than those of physicians (17% agreeing to a greater or lesser extent). Most participants disagreed with the need to consider treatment costs (53%), resource allocation matters (54%), and the need for vacant ICU beds (91%) in this context.

Nurses considered treatment costs (*U* = 1,508.5; *p* = 0.092), resource allocation, and need for free ICU beds (*U* = 1,623; *p* = 0.004) more relevant than did physicians, while physicians emphasized more the legal regulations (*U* = 988.5; *p* = 0.066) and the importance of the patient's interests (*U* = 1,004; *p* = 0.054). In addition, physicians were generally more prone than nurses to consider religious and cultural determinants as important (for everyone involved), although this finding did not reach statistical differences.

A more detailed list of participants’ responses is shown in Table 4.

**Table 4:** Major considerations of healthcare professionals regarding LST limitation at designated ICUs: description and analysis according to vocation (nurses/physicians).

Question	Answer	All, *N* (%)	Physician, *N* (%)	Nurse, *N* (%)	Mann–Whitney	
					*U*	*p*
III.9.1. Good medical practice	0^a^	1 (0.9%)	0 (0.0%)	1 (1.2%)	1,153.000	0.447
	Strongly disagree	2 (1.8%)	0 (0.0%)	2 (2.4%)		
	Disagree	3 (2.7%)	1 (3.2%)	2 (2.4%)		
	Undecided	7 (6.2%)	1 (3.2%)	6 (7.3%)		
	Agree	33 (29.2%)	9 (29.0%)	24 (29.3%)		
	Strongly agree	67 (59.3%)	20 (64.5%)	47 (57.3%)		
III.9.2. Patient's interest	0^a^	1 (0.9%)	0 (0.0%)	1 (1.2%)	1,004.000	0.054
	Disagree	1 (0.9%)	0 (0.0%)	1 (1.2%)		
	Undecided	7 (6.2%)	1 (3.2%)	6 (7.3%)		
	Agree	33 (29.2%)	6 (19.4%)	27 (32.9%)		
	Strongly agree	71 (62.8%)	24 (77.4%)	47 (57.3%)		
III.9.3. Patient's autonomy	0^a^	1 (0.9%)	0 (0.0%)	1 (1.2%)	1,121.000	0.348
	Strongly disagree	1 (0.9%)	0 (0.0%)	1 (1.2%)		
	Disagree	1 (0.9%)	0 (0.0%)	1 (1.2%)		
	Undecided	20 (17.7%)	4 (12.9%)	16 (19.5%)		
	Agree	41 (36.3%)	12 (38.7%)	29 (35.4%)		
	Strongly agree	49 (43.4%)	15 (48.4%)	34 (41.5%)		
III.9.4. Treatment costs	0^a^	1 (0.9%)	0 (0.0%)	1 (1.2%)	1,508.500	0.092
	Strongly disagree	25 (22.1%)	8 (25.8%)	17 (20.7%)		
	Disagree	28 (24.8%)	11 (35.5%)	17 (20.7%)		
	Undecided	23 (20.4%)	6 (19.4%)	17 (20.7%)		
	Agree	25 (22.1%)	4 (12.9%)	21 (25.6%)		
	Strongly agree	11 (9.7%)	2 (6.5%)	9 (11.0%)		
III.9.5. ACDs	0^a^	3 (2.7%)	1 (3.2%)	2 (2.4%)	1,232.000	0.817
	Strongly disagree	1 (0.9%)	0 (0.0%)	1 (1.2%)		
	Disagree	1 (0.9%)	0 (0.0%)	1 (1.2%)		
	Undecided	13 (11.5%)	4 (12.9%)	9 (11.0%)		
	Agree	48 (42.5%)	14 (45.2%)	34 (41.5%)		
	Strongly agree	47 (41.6%)	12 (38.7%)	35 (42.7%)		
III.9.6. Wishes of family/legal representatives	Disagree	10 (8.8%)	4 (12.9%)	6 (7.3%)	1,310.000	0.789
	Undecided	31 (27.4%)	7 (22.6%)	24 (29.3%)		
	Agree	55 (48.7%)	16 (51.6%)	39 (47.6%)		
	Strongly agree	17 (15.0%)	4 (12.9%)	13 (15.9%)		
III.9.7. Legal regulations	0^a^	1 (0.9%)	0 (0.0%)	1 (1.2%)	988.500	0.066
	Strongly disagree	3 (2.7%)	0 (0.0%)	3 (3.7%)		
	Disagree	5 (4.4%)	2 (6.5%)	3 (3.7%)		
	Undecided	24 (21.2%)	2 (6.5%)	22 (26.8%)		
	Agree	48 (42.5%)	16 (51.6%)	32 (39.0%)		
	Strongly agree	32 (28.3%)	11 (35.5%)	21 (25.6%)		
III.9.8. Religious principles	0^a^	1 (0.9%)	0 (0.0%)	1 (1.2%)	1,162.500	0.533
	Strongly disagree	8 (7.1%)	0 (0.0%)	8 (9.8%)		
	Disagree	20 (17.7%)	7 (22.6%)	13 (15.9%)		
	Undecided	38 (33.6%)	10 (32.3%)	28 (34.1%)		
	Agree	31 (27.4%)	10 (32.3%)	21 (25.6%)		
	Strongly agree	15 (13.3%)	4 (12.9%)	11 (13.4%)		
III.9.9. Need for vacant ICU beds	0^a^	2 (1.8%)	0 (0.0%)	2 (2.4%)	1,623.000	0.004
	Strongly disagree	66 (58.4%)	25 (80.6%)	41 (50.0%)		
	Disagree	25 (22.1%)	4 (12.9%)	21 (25.6%)		
	Undecided	9 (8.0%)	1 (3.2%)	8 (9.8%)		
	Agree	6 (5.3%)	1 (3.2%)	5 (6.1%)		
	Strongly agree	5 (4.4%)	0 (0.0%)	5 (6.1%)		
III.11. Resource allocation considerations	0^a^	4 (3.5%)	1 (3.2%)	3 (3.7%)	1,319.500	0.347
	Not important at all	32 (28.3%)	9 (29.0%)	23 (28.0%)		
	Not important	29 (25.7%)	11 (35.5%)	18 (22.0%)		
	Undecided	29 (25.7%)	6 (19.4%)	23 (28.0%)		
	Important	16 (14.2%)	4 (12.9%)	12 (14.6%)		
	Very important	3 (2.7%)	0 (0.0%)	3 (3.7%)		
III.14. Different religious/cultural beliefs of patient or legal representative	0^a^	1 (0.9%)	0 (0.0%)	1 (1.2%)	1,379.000	0.378
	Strongly disagree	2 (1.8%)	0 (0.0%)	2 (2.4%)		
	Disagree	8 (7.1%)	4 (12.9%)	4 (4.9%)		
	Undecided	35 (31.0%)	9 (29.0%)	26 (31.7%)		
	Agree	59 (52.2%)	18 (58.1%)	41 (50.0%)		
	Strongly agree	8 (7.1%)	0 (0.0%)	8 (9.8%)		
III.15. Different religious/cultural beliefs of physician	0^a^	2 (1.8%)	0 (0.0%)	2 (2.4%)	1,398.500	0.281
	Strongly disagree	34 (30.1%)	14 (45.2%)	20 (24.4%)		
	Disagree	34 (30.1%)	6 (19.4%)	28 (34.1%)		
	Undecided	23 (20.4%)	4 (12.9%)	19 (23.2%)		
	Agree	18 (15.9%)	7 (22.6%)	11 (13.4%)		
	Strongly agree	2 (1.8%)	0 (0.0%)	2 (2.4%)		

^a^ 0 = Non-response to a given questionnaire item.

### 4.4 Analysis by regional and professional experience differences

#### 4.4.1 Regional differences

Healthcare professionals from Rijeka reported significantly greater experience with DNACPR decisions than those from Zagreb (*p* = 0.002) and more experience with LST withholding than those from Split (*p* = 0.034). There was no difference in participants’ experience with the withdrawal of various procedures (namely, mechanical ventilation, inotropes, and hydration) apart from antibiotics withdrawal, which was more often encountered in Split than in Zagreb (*p* = 0.034).

Recording of DNACPR orders is more often in Rijeka than both in Zagreb (*p* = 0.019) and Split (*p* = 0.010), while compliance with these orders is greater in Rijeka than in Split (*p* = 0.045). Participants from Split were less likely to report a lack of consensus among physicians (*p* < 0.001 compared to Zagreb; *p* = 0.019 compared to Rijeka). Professionals from Rijeka were more aware of whom to contact for ethical dilemmas (*p* < 0.001 than Zagreb; *p* = 0.021 than Split) and more often consulted ethics committees (*p* = 0.009 than Zagreb; *p* = 0.008 than Split).

Participants from both Rijeka (*p* = 0.007; *p* = 0.021) and Split (*p* = 0.026, *p* = 0.003) were more likely to consider the limitation of LST and DNACPR as ethically acceptable than those from Zagreb. Participants from Split viewed the limitation of hydration as acceptable and highlighted their individual responsibility more often than participants from Rijeka (in both cases, *p* = 0.020).

Healthcare providers from Rijeka were more likely than providers from both Zagreb (*p* < 0.001; *p* = 0.001) and Split (*p* = 0.013; *p* = 0.029) to highlight treatment costs and the need for free ICU beds as important considerations. In relation to participants from Zagreb, participants from Rijeka were also more likely to highlight the importance of wishes from family members (and legal representatives) (*p* = 0.008), religious principles (*p* = 0.011), physicians’ religious and cultural beliefs (*p* = 0.003), and resource allocation considerations (*p* = 0.013). However, professionals from Zagreb placed a greater emphasis on legal regulations than Rijeka (*p* = 0.006) and less on patients’ or legal representatives’ religious and cultural beliefs than professionals from both Rijeka (*p* = 0.006) and Split (*p* = 0.002).

A more detailed list of responses by regional differences is available in the Supplementary Material (Supplementary Table 1).

#### 4.4.2 Professional experience differences

More experienced healthcare professionals were more aware of whom to contact when encountering ethical dilemmas (*p* = 0.012), were less likely to comply with DNACPR orders (*p* = 0.003), highlighted the importance of legal regulations (*p* = 0.048), highlighted the need to respect different religious/cultural beliefs of patients or representatives (*p* = 0.014), perceive adult and pediatric LST limitation context as equal (*p* = 0.048), and consider ACD as a useful tool (*p* = 0.004).

A more detailed list of responses by professional experience differences is available in the Supplementary Material (Supplementary Table 2).

## 5 Discussion

This study represents the investigation into the experiences, attitudes, and significant considerations of professionals in Croatian pediatric ICUs regarding end-of-life care. Several main findings can be drawn from this study, and they will be discussed further.

### 5.1 Commonness and views on ethics of LST limitation in Croatian pediatric ICUs

The findings indicate that limitations of LST, including both withdrawing and withholding, are considerably less common than in other countries. For example, in “Western” high-income countries, 40%–70% of deaths within pediatric ICUs occur after withholding or withdrawing LSTs (3, 5, 6, 27). These findings align with previous findings from a survey of healthcare professionals from adult ICUs in Croatia (22).

However, it seems that this trend is even more prominent in Croatian pediatric ICUs as DNACPR, withholding, and, to a lesser extent, LST withdrawing are even less common than in Croatian adult ICUs for example, 40% vs. 19.2% for DNACPR and 36.3% vs. 24.2% for withholding (22). This is a rather interesting finding, as such a difference between adult and pediatric ICUs was not to be expected based on findings from previous studies from (3, 6, 13, 14) for pediatric and (27–29) for adult settings.

There is an unusual contrast between Croatian adult and pediatric ICU professionals also regarding their views on the ethical acceptability of DNACPR and LST limitations. Only approximately half of pediatric ICU professionals view these practices as acceptable compared to two-thirds in adult ICUs (22). When compared to findings from other countries, these findings are pretty surprising. For example, a vast majority of Slovene pediatricians consider the limitation of LST as ethically acceptable (26), and similar findings can be found elsewhere (12, 30, 31).

Nonetheless, Croatian adult and pediatric ICU professionals share their ambivalence when evaluating ethical differences between withholding and withdrawing LST (22). The nearly equal split of opinions, contrary to the most common ethical standpoints (7, 32), mirrors the findings from other similar studies (26, 31).

### 5.2 Possible reasons for differences found between Croatian adult and pediatric ICU settings

Previously outlined findings, putting aside possible broader influences, might reflect the fact that there are indeed some significant differences between adult and pediatric end-of-life contexts. Pediatric patients are generally more diverse and complex while also less able to exercise autonomy. This makes healthcare providers more reliant on proxy decision-makers but also highlights the imperative of the primacy of patients’ best interests (33, 34). However, determining the patient's best interest might be quite ambiguous, especially in complex cases burdened with prognostic uncertainty when possible treatments often fall between being clearly beneficial and clearly futile (24, 34). Recent studies also indicate essential differences in end-of-life care considerations between different types of pediatric ICUs—between NICUs and PICUs, as decisions in NICUs are more often based on quality-of-life while in PICUs on survival likelihood considerations (33, 35, 36). Interestingly enough, and quite contrary to what was just mentioned, participants from this study were not likely to characterize differences between adult and pediatric end-of-life contexts as decisive.

### 5.3 Possible reasons for lower acceptance rates and lesser experience with LST limitation practices in Croatia

Empirical evidence stresses the importance of many influences on end-of-life perspectives and practices. Besides those case or patient, patient representative, and healthcare provider related, they also include organizational, cultural, and, more broadly speaking, social (21, 31, 37, 38). As mentioned, several recent studies on end-of-life issues in Croatia outlined somewhat more “traditional” views compared to other countries. It's important to emphasize that the term “traditional” is not intended to be pejorative but rather to highlight the connection to more classical and even Hippocratic perspectives on the discussed issues. Studies on the public revealed a rising but still quite low level of acceptance (21). These views are significantly influenced by factors such as age, education, place of residence, and political orientation. In short, younger, more educated, more liberal in their political orientation, and those from urban settings showed a greater level of acceptance of LST limitation as well as euthanasia and assisted suicide (21). Croatian ICU healthcare professionals showed a greater level of acceptance, but both levels of acceptance and actual experience with the limitation of LST are still lower than in other countries (22). Possible reasons already stated from previous Croatian studies are more paternalistic and conservative predilections, ambiguity and vagueness of legal context, and lack of clinical and professional guidelines (21–24, 39). Similarly, Devictor et al. (40), based on findings from landmark pediatric end-of-life studies, also highlighted possible influences of broader ex-communist Eastern European sociocultural factors. This study strongly reaffirms all previous findings while, as previously discussed, adding an additional layer of complexity due to observed marked differences between adult and pediatric contexts.

### 5.4 Decision-makers and decision-making values and processes

Family members or other patient representatives are frequently involved in LST limitation decision-making processes, similar to findings from many other studies (31, 41, 42). However, healthcare professionals are rarely informed about proxy decision-makers’ wishes, and ACDs, although valued, are seldom encountered. These findings combined point to the possible challenges of the true involvement of both patients and their representatives in decision-making. This might be reminiscent of a more general ambiguity of legal context, as surveyed professionals generally emphasized the importance of the principle of respecting autonomy. On the other hand, specific paternalistic stances of healthcare professionals can also be here at play, as already illustrated by previous studies within the Croatian healthcare context (43, 44). Indeed, when evaluating most important end-of-life considerations, surveyed professionals placed a somewhat higher value on patients’ best interest and good medical practice than on the respect for autonomy. In addition, although generally rare, a lack of consensus was more often perceived between patient representatives and physicians as primary discussion initiators and decision-makers. All the above may be further complicated by expected limited health literacy in the Croatian population, disproportionally affecting those already “worse off” (45). This was also evident from a previous qualitative study in the same settings, where Croatian healthcare professionals highlighted barriers to patient representatives' true awareness, informedness, and disagreements, especially regarding the futility of certain procedures (24). Of note, the Family Act, the primary regulation concerning professional-child-parent/representative interactions in the context of Croatian healthcare, allows special non-litigious court procedures aimed at protecting a child's welfare when discrepancies in opinion between critical decision-makers (child aged over 16 years, representative, or physician) are present.

Nonetheless, nurses in Croatian pediatric ICUs are strikingly less involved in LST limitation decision-making, including initiation and contribution to discussions. This finding aligns with similar ones elsewhere but raises concerns given the crucial role of nurses in end-of-life care (12, 31, 46–48). Previous qualitative research in the same setting indicated that nurses emphasized interprofessional relationships, while physicians were focused more on intra-professional dynamics (24). The same study also found nurses’ contributions to be “highly valued, facilitated, and appreciated” (24). However, other studies in a Croatian healthcare context, as well as elsewhere, also pointed out a rather strict internal (between different medical specializations) and external (between different healthcare professionals) hierarchical structure, contributing to numerous challenges on the many different levels of the healthcare system (49). This is of great importance as discrepancies between perceptions of nurses and physicians are, despite not being uncommon, identified as one of the greatest barriers in delivering appropriate end-of-life care (14, 50).

When decisions in Croatian pediatric ICUs are finally reached, they are enacted almost immediately but without the presence of any written instructions, as they are either absent or only verbal. These particular findings, while reaffirming previous ones, might be a reflection of the lack of not only clear national guidelines but also policies, that is, legal regulations (22, 24, 51).

### 5.5 Differences by region within Croatia: possible explanations

A significant finding of this study is great variability among different regions, represented by major cities: Zagreb, Rijeka, and Split, both in terms of experiences with, attitudes towards, and primary considerations underpinning LST limitation. If the most relevant regional differences are summed up, professionals from Rijeka have greater acceptance and more experience with LST limitation practices than those from Split and Zagreb.

This is in line with previous local and global findings. Previous research in Croatia on end-of-life issues signaled significant variability in decision-making determinants, processes, and related practices (22–24), and similar findings can also be found elsewhere. High variability is quite certainly a reflection of the complexity of the issue at hand, being influenced by numerous, more or less proximal, factors, but is also a quite straightforward argument for actions aimed at optimization (31, 37, 38). Findings on high variability from this study as Croatia is a relatively small country, with a population, according to the last Census in 2021, of 4,047,680 residents with a relatively ethnically homogenous structure (16, 21), might point toward the importance of organizational/institutional factors. This may also be supported by findings from survey on Croatian adult ICU professionals, which showed significant influences of types of ICUs and their broader hospital context on end-of-life practices (22). Another possible explanation might be the more nuanced social and cultural differences between these regions. However, even in cultural terms, Croatia seems to be rather homogenous—differences are not sufficient to form distinct regional profiles, while regions themselves are not well defined only by geographical characteristics (52). Nonetheless, more experience with and higher acceptance of LST limitation by professionals from Rijeka might potentially be explained by relative proximity, in an actual and metaphoric sense, to Italy and Slovenia, as their perspectives on end-of-life issues seem to be more inclined to those of their fellow neighbors and “Western” countries in general.

### 5.6 “Meta-finding” of high ambiguity

Another important “meta-finding” from this study, also present in similar studies performed in Croatia including both professionals and the public (21, 22), is a notable share of responses that reflect profound ambivalences regarding end-of-life issues. This ambivalence, seen in high rates of “undecided” answers, may stem from the broader sociocultural context (15, 53). After all, it is certainly not a matter of mere coincidence that in Croatia, the established legal frame for withholding and withdrawing is still non-existent. It is as if all are turning a blind eye, failing to recognize that sometimes one can do more harm than good simply by doing everything one can do. By its advances, medicine allows many great things, many of which have recently been unprecedented. However, that does not automatically mean that these “things” are always good nor beneficial. They can also be futile, utterly disproportionate in terms of expected outcomes. However, and here lies the great trouble; such interventions can also lie somewhere in between. The only way to determine their value in some instances and contexts is through truly shared decision-making (34, 42, 54–56). This is then the only proper way to make not only the right, but also the good decision, one with which each of the persons involved can be, at best, satisfied while at least at peace (49, 55, 57).

### 5.7 A possible way forward

Some of the previously emphasized Croatia specific influences are more or less modifiable. What seems to be the easiest solution, while clearly within professional responsibilities, is the creation of comprehensive end-of-life guidelines.

The recent publication of “Guidelines for improving quality of palliative care in intensive medicine” by the Ministry of Healthcare in Croatia is undoubtedly a positive step (available at: https://zdravstvo.gov.hr/UserDocsImages/dokumenti/Tekstovi%20razni/Smjernice%20za%20unaprje%C4%91enje%20kvalitete%20palijativne%20skrbi%20u%20intenzivnoj%20medicini_online%20verzija.pdf). However, even here, one can sense a great caution in this (“top-down”) approach by examining the guidelines’ very title. It may come as no surprise that most physicians (intensivists) still do not apply these guidelines, mainly due to the fears stemming from the lack of legal protection and misperceptions from colleagues and family members (51).

This again stresses the importance of the broader legal framework, also a factor that is, at least in principle, modifiable, while also, one could argue, falling within professional responsibilities (49). However, the legal system is largely reflective of a broader sociocultural context. Then again, a lot still needs to be done, primarily in terms of raising the overall health literacy. These are all reinforcing issues, as physicians are less likely to give patients more authority in a setting without clear legal regulations (31).

Due to widespread possibilities of misperceptions, support mechanisms should be implemented, especially as a previous study emphasized the high burden associated with pediatric end-of-life care in this setting, having a great influence both on the personal and professional lives of professionals (24).

Nevertheless, given many “unknowns” are still present, further exploration of fine nuances influencing this critical aspect of pediatric care is needed, as their exploration is crucial for tailoring intervention and policies to this rather specific local context.

### 5.8 Limitations

This study used a quantitative method—a questionnaire—adopted from previous Slovenian studies, slightly adjusted, and pilot-tested. In that sense, limitations are all those related using such methodology, just to name a few: lack of in-depth insights, recency bias, and social desirability bias. The latter might be of great importance here as the topic is, as many of the findings also suggest, still rather controversial. That is why we used a specific method of approaching potential participants (through immediate colleagues) and emphasized anonymity. In addition, as mentioned earlier, this study is part of the larger research project and, as such, complements findings from other sources and those collected through other research means and methodologies.

Another limitation is the nature of sampling, which could introduce selection bias. In addition, the generalizability of findings from this study might be limited because one potential site (University Clinical Hospital Osijek) did not participate. Further, the response rate, especially in the nurses’ section, is rather low. There are, however, good reasons for that. First, we calculated the response rate in relation to the total eligible population of healthcare professionals—all those working in Croatian pediatric ICUs except Osijek. In other words, not all eligible population members were actually reached (for example, because of absence from work during the study period). Nurses also work in shifts more often, covering the whole day, which could have made them less reachable. Therefore, there is a significant gap between those eligible healthcare professionals and those who were included. All the above makes the yielded low response rate more tolerable (as it is, in essence, underestimated) with a more likely non-systematic (than systemic) influence on measured outcomes.

## 6 Conclusion

This study has illuminated the complexities of end-of-life care practices in pediatric intensive care units in Croatia. Through a comprehensive survey of healthcare professionals, valuable insights into the experiences, challenges, and primary ethical considerations faced by those at the very frontline of pediatric intensive care are provided. The picture it sketched could be better. It reaffirms significant variability in end-of-life decision-making processes and practices from previous studies. It also signals profound ambiguity of healthcare professionals’ related attitudes and beliefs and a need for mote experience with and involvement in such discussions and practices, especially on the part of nurses. The findings underscore the need for clearer guidelines, legal frameworks, improved support mechanisms, and enhanced communication strategies to navigate the complex ethical challenges of pediatric end-of-life care and decision-making. As Croatia moves toward establishing more defined policies, this research contributes to a deeper understanding of the nuanced nature of pediatric end-of-life, advocating for practices that prioritize compassionate, patient- and family-centered care, the wellbeing of everyone included, while above all, the professionals’ integrity and patients’ dignity.

## Data Availability

The original contributions presented in the study are included in the article/Supplementary Material, further inquiries can be directed to the corresponding author.

## References

[1] Pousset G Bilsen J Cohen J Addington-Hall J Miccinesi G Onwuteaka-Philipsen B Deaths of children occurring at home in six European countries. Child Care Health Dev. (2010) 36(3):375–84. 10.1111/j.1365-2214.2009.01028.x 19961493

[2] Chang E MacLeod R Drake R. Characteristics influencing location of death for children with life-limiting illness. Arch Dis Child. (2013) 98(6):419–24. 10.1136/archdischild-2012-301893 23599439

[3] Burns JP Sellers DE Meyer EC Lewis-Newby M Truog RD. Epidemiology of death in the PICU at five U.S. teaching hospitals. Crit Care Med. (2014) 42(9):2101–8. 10.1097/CCM.0000000000000498 24979486 PMC4134743

[4] Meert KL Keele L Morrison W Berg RA Dalton H Newth CJL End-of-life practices among tertiary care PICUs in the United States: a multicenter study. Pediatr Crit Care Med. (2015) 16:7. 10.1097/PCC.0000000000000520 PMC4562059 26335128

[5] Roth A Rapoport A Widger K Friedman JN. General paediatric inpatient deaths over a 15-year period. Paediatr Child Health. (2017) 22:2. 10.1093/pch/pxx005 29479186 PMC5804955

[6] Trowbridge A Walter JK McConathey E Morrison W Feudtner C. Modes of death within a children’s hospital. Pediatrics. (2018) 142(4):e20174182. 10.1542/peds.2017-4182 30232217

[7] Sprung CL Truog RD Curtis JR Joynt GM Baras M Michalsen A Seeking worldwide professional consensus on the principles of end-of-life care for the critically ill: the consensus for worldwide end-of-life practice for patients in intensive care units (WELPICUS) study. Am J Respir Crit Care Med. (2014) 190(8):855–66. 10.1164/rccm.201403-0593CC 25162767

[8] Orioles A Morrison WE. Medical ethics in pediatric critical care. Crit Care Clin. (2013) 29(2):359–75. 10.1016/j.ccc.2012.12.002 23537680

[9] Buang SNH Loh SW Mok YH Lee JH Chan YH. Palliative and critical care: their convergence in the pediatric intensive care unit. Front Pediatr. (2022) 10:907268. 10.3389/fped.2022.907268 35757116 PMC9226486

[10] Sieg SE Bradshaw WT Blake S. The best interests of infants and families during palliative care at the end of life: a review of the literature. Adv Neonatal Care. (2019) 19(2):E9–14. 10.1097/ANC.0000000000000567 30394915

[11] Zhong Y Cavolo A Labarque V Gastmans C. Physician decision-making process about withholding/withdrawing life-sustaining treatments in paediatric patients: a systematic review of qualitative evidence. BMC Palliat Care. (2022) 21(1):113. 10.1186/s12904-022-01003-5 35751075 PMC9229823

[12] Burns JP Mitchell C Griffith JL Truog RD. End-of-life care in the pediatric intensive care unit: attitudes and practices of pediatric critical care physicians and nurses. Crit Care Med. (2001) 29(3):658–64. 10.1097/00003246-200103000-00036 11373439

[13] Devictor DJ Nguyen DT. Forgoing life-sustaining treatments in children: a comparison between northern and Southern European pediatric intensive care units. Pediatr Crit Care Med. (2004) 5(3):211–5. 10.1097/01.PCC.0000123553.22405.E3 15115556

[14] Devictor DJ Latour JM, EURYDICE II Study Group. Forgoing life support: how the decision is made in European pediatric intensive care units. Intensive Care Med. (2011) 37(11):1881–7. 10.1007/s00134-011-2357-3 21965096

[15] Kirsch RE Balit CR Carnevale FA Latour JM Larcher V. Ethical, cultural, social, and individual considerations prior to transition to limitation or withdrawal of life-sustaining therapies. Pediatr Crit Care Med. (2018) 19(8S Suppl 2):S10–8. 10.1097/PCC.0000000000001488 30080802

[16] Nikodem K Ćurković M Borovečki A. Trust in the healthcare system and physicians in Croatia: a survey of the general population. Int J Environ Res Public Health. (2022) 19(2):993. 10.3390/ijerph19020993 35055815 PMC8796022

[17] Croatian Bureau of Statistics. Population by Ethnicity and Religion, 2021 Census. Statistics in Line (2023). Available online at: https://podaci.dzs.hr/en/statistics/population/ (accessed June 20, 2024).

[18] Croatian Bureau of Statistics. Average age of population, 1953–2021 Censuses. Statistics in Line (2023). Available online at: https://podaci.dzs.hr/en/statistics/population/ (accessed June 20, 2024).

[19] Hodžić S Vuković D Muharemović A. The efficiency of healthcare system expenditures: evidence from Croatia. Ekon Vjesn. (2019) 32:361–71.

[20] OECD/European Observatory on Health Systems and Policies. Croatia: Country Health Profile 2017. Brussels: State of Health in the EU, OECD Publishing, Paris/European Observatory on Health Systems and Policies (2017). 10.1787/9789264283312-en

[21] Borovecki A Curkovic M Nikodem K Oreskovic S Novak M Rubic F Attitudes about withholding or withdrawing life-prolonging treatment, euthanasia, assisted suicide, and physician assisted suicide: a cross-sectional survey among the general public in Croatia. BMC Med Ethics. (2022) 23(1):13. 10.1186/s12910-022-00751-6 35172812 PMC8851732

[22] Špoljar D Vučić M Peršec J Merc V Kereš T Radonić R Experiences and attitudes of medical professionals on treatment of end-of-life patients in intensive care units in the republic of Croatia: a cross-sectional study. BMC Med Ethics. (2022) 23(1):12. 10.1186/s12910-022-00752-5 35172834 PMC8851755

[23] Ćurković M Brajković L Jozepović A Tonković D Župan Ž Karanović N End-of-life decisions in intensive care units in Croatia—pre COVID-19 perspectives and experiences from nurses and physicians. J Bioeth Inq. (2021) 18(4):629–43. 10.1007/s11673-021-10128-w 34554388 PMC8459337

[24] Rubic F Curkovic M Brajkovic L Nevajdic B Novak M Filipovic-Grcic B End-of-life decision-making in pediatric and neonatal intensive care units in Croatia—a focus group study among nurses and physicians. Medicina (Kaunas). (2022) 58(2):250. 10.3390/medicina58020250 35208575 PMC8879945

[25] Groselj U Orazem M Kanic M Vidmar G Grosek S. Experiences of Slovene ICU physicians with end-of-life decision making: a nation-wide survey. Med Sci Monit. (2014) 20:2007–12. 10.12659/MSM.891029 25335864 PMC4214698

[26] Grosek S Orazem M Kanic M Vidmar G Groselj U. Attitudes of Slovene paediatricians to end-of-life care. J Paediatr Child Health. (2016) 52(3):278–83. 10.1111/jpc.13006 26515146

[27] Wanders A Ghinescu C Levy-Jamet Y Martin A-L Barcos-Munoz F Rimensberger P Circumstances surrounding end of life in a Swiss pediatric intensive care unit. Intensive Care Med. (2023) 112(5):e371. 10.1007/s44253-023-00005-2

[28] Sprung CL Cohen SL Sjokvist P Baras M Bulow HH Hovilehto S End-of-life practices in European intensive care units: the Ethicus Study. JAMA. (2003) 290(6):790–7. 10.1001/jama.290.6.790 12915432

[29] Sprung CL Ricou B Hartog CS Maia P Mentzelopoulos SD Weiss M Changes in end-of-life practices in European intensive care units from 1999 to 2016. JAMA. (2019) 322(17):1692–704. 10.1001/jama.2019.14608 31577037 PMC6777263

[30] Sanchez Varela AM Johnson LM Kane JR Kasow KA Quintana Y Coan A Ethical decision making about end-of-life care issues by pediatric oncologists in economically diverse settings. J Pediatr Hematol Oncol. (2015) 37(4):257–63. 10.1097/MPH.0000000000000271 25887639

[31] Zhong Y Cavolo A Labarque V Gastmans C. Physicians’ attitudes and experiences about withholding/withdrawing life-sustaining treatments in pediatrics: a systematic review of quantitative evidence. BMC Palliat Care. (2023) 22(1):145. 10.1186/s12904-023-01260-y 37773128 PMC10540364

[32] Spoljar D Curkovic M Gastmans C Gordijn B Vrkic D Jozepovic A Ethical content of expert recommendations for end-of-life decision-making in intensive care units: a systematic review. J Crit Care. (2020) 58:10–9. 10.1016/j.jcrc.2020.03.010 32278227

[33] Fontana MS Farrell C Gauvin F Lacroix J Janvier A. Modes of death in pediatrics: differences in the ethical approach in neonatal and pediatric patients. J Pediatr. (2013) 162(6):1107–11. 10.1016/j.jpeds.2012.12.008 23312685

[34] Lantos JD. Ethical problems in decision making in the neonatal ICU. N Engl J Med. (2018) 379(19):1851–60. 10.1056/NEJMra1801063 30403936

[35] Larcher V Craig F Bhogal K Wilkinson D Brierley J, Royal College of Paediatrics and Child Health. Making decisions to limit treatment in life-limiting and life-threatening conditions in children: a framework for practice. Arch Dis Child. (2015) 100(Suppl 2):s3–23. 10.1136/archdischild-2014-306666 25802250

[36] Snoep MC Jansen NJG Groenendaal F. Deaths and end-of-life decisions differed between neonatal and paediatric intensive care units at the same children’s hospital. Acta Paediatr. (2018) 107(2):270–5. 10.1111/apa.14061 28871637 PMC5813263

[37] Meñaca A Evans N Andrew EV Toscani F Finetti S Gómez-Batiste X End-of-life care across Southern Europe: a critical review of cultural similarities and differences between Italy, Spain and Portugal. Crit Rev Oncol Hematol. (2012) 82(3):387–401. 10.1016/j.critrevonc.2011.06.002 21741855

[38] Mark NM Rayner SG Lee NJ Curtis JR. Global variability in withholding and withdrawal of life-sustaining treatment in the intensive care unit: a systematic review. Intensive Care Med. (2015) 41(9):1572–85. 10.1007/s00134-015-3810-5 25904183

[39] Borovečki A Nikodem K Ćurković M Braš M Palić-Kramarić R Špoljar D What constitutes a “good death”?—a representative cross-sectional survey among the general public in Croatia. Omega (Westport). (2023) 86(4):1415–31. 10.1177/00302228211010597 33940964

[40] Devictor DJ Tissieres P Gillis J Truog R. Intercontinental differences in end-of-life attitudes in the pediatric intensive care unit: results of a worldwide survey. Pediatr Crit Care Med. (2008) 9(6):560–6. 10.1097/PCC.0b013e31818d358 18838925

[41] Akkermans AA Lamerichs JMWJJ Schultz MJM Cherpanath TGVT van Woensel JBMJ van Heerde MM How doctors actually (do not) involve families in decisions to continue or discontinue life-sustaining treatment in neonatal, pediatric, and adult intensive care: a qualitative study. Palliat Med. (2021) 35(10):1865–77. 10.1177/02692163211028079 34176357 PMC8637379

[42] Gillam L Sullivan J. Ethics at the end of life: who should make decisions about treatment limitation for young children with life-threatening or life-limiting conditions? J Paediatr Child Health. (2011) 47(9):594–8. 10.1111/j.1440-1754.2011.02177.x 21951439

[43] Vučemilo L Ćurković M Milošević M Mustajbegović J Borovečki A. Are physician-patient communication practices slowly changing in Croatia?—a cross-sectional questionnaire study. Croat Med J. (2013) 54(2):185–91. 10.3325/cmj.2013.54.185 23630146 PMC3641876

[44] Vučemilo L Borovečki A. Readability and content assessment of informed consent forms for medical procedures in Croatia. PLoS One. (2015) 10(9):e0138017. 10.1371/journal.pone.0138017 26376183 PMC4573755

[45] Bobinac A. Access to healthcare and health literacy in Croatia: empirical investigation. Healthcare (Basel). (2023) 11(13):1955. 10.3390/healthcare11131955 37444789 PMC10340794

[46] Keenan HT Diekema DS O'Rourke PP Cummings P Woodrum DE. Attitudes toward limitation of support in a pediatric intensive care unit. Crit Care Med. (2000) 28(5):1590–4. 10.1097/00003246-200005000-00055 10834717

[47] Beckstrand RL Willmore EE Macintosh JLB Luthy KEB. Critical care nurses’ qualitative reports of experiences with physician behaviors, nursing issues, and other obstacles in end-of-life care. Dimens Crit Care Nurs. (2021) 40(4):237–47. 10.1097/DCC.0000000000000479 34033445

[48] Zanin A Brierley J Latour JM Gawronski O. End-of-life decisions and practices as viewed by health professionals in pediatric critical care: a European survey study. Front Pediatr. (2023) 10:1067860. 10.3389/fped.2022.1067860 36704131 PMC9872024

[49] Ćurković M Borovecki A. The Bridge Between Bioethics and Medical Practice: Medical Professionalism. Cham: Springer (2022).

[50] Keele L Meert KL Berg RA Dalton H Newth CJL Harrison R Limiting and withdrawing life support in the PICU: for whom are these options discussed? Pediatr Crit Care Med. (2016) 17:2. 10.1097/PCC.0000000000000614 26669647 PMC5029280

[51] Leventić V Nešković N Kvolik S Kristek G Škiljić S Haršanji-Drenjančević I. Are we ready for end of life decisions in intensive medicine? Liječnički Vjesnik. (2023) 145(Suppl 4):60–6. (in Croatian). 10.26800/lv-145-supl4-14

[52] Rajh E Budak J Anić ID. Hofstede’s culture value survey in Croatia: examining regional differences. Društvena Istraživanja. (2016) 25:309–27. 10.5559/di.25.3.02

[53] Gómez-Vírseda C de Maeseneer Y Gastmans C. Relational autonomy in end-of-life care ethics: a contextualized approach to real-life complexities. BMC Med Ethics. (2020) 21(1):50. 10.1186/s12910-020-00495-1 32605569 PMC7325052

[54] Davidson JE Aslakson RA Long AC Puntillo KA Kross EK Hart J Guidelines for family-centered care in the neonatal, pediatric, and adult ICU. Crit Care Med. (2017) 45(1):103–28. 10.1097/CCM.0000000000002169 27984278

[55] Gómez-Vírseda C de Maeseneer Y Gastmans C. Relational autonomy: what does it mean and how is it used in end-of-life care? A systematic review of argument-based ethics literature. BMC Med Ethics. (2019) 20(1):76. 10.1186/s12910-019-0417-3 31655573 PMC6815421

[56] Linebarger JS Johnson V Boss RD Linebarger JS Collura CA Humphrey LM Guidance for pediatric end-of-life care. Pediatrics. (2022) 149(5):e2022057011. 10.1542/peds.2022-057011 35490287

[57] Curkovic M Kosec A. The ethics (mis)used for filling the voids or harm of harm reduction ethics. J Geriatr Oncol. (2020) 11(7):1168–9. 10.1016/j.jgo.2020.05.002 32418880 PMC7205650

