# Supplementary material for: Navigating the shadows: medical professionals’ values and perspectives on end-of-life care within pediatric intensive care units in Croatia
Source: Front Pediatr. 2024 Aug 12;12:1394071. doi: 10.3389/fped.2024.1394071 (PMC11345198; doi:10.3389/fped.2024.1394071)
Supplement: Supplementary file 1 [file Table1.pdf]

Supplementary Table 1. Experiences, attitudes and values of Croatian pediatric ICU healthcare professionals regarding LST limitation – analysis by region

	Sum of Squares	F	p		N	Mean	SD	Post Hoc Comparisons		Mean Difference	SE	t	p_{tukey}
**I.11. Awareness of Contact for Ethical Dilemma**	4.401	11.411	< .001	Rijeka	22	1.273	0.456	Rijeka	Split	-0.352	0.130	-2.718	0.021
				Split	24	1.625	0.495		Zagreb	-0.515	0.108	-4.765	< .001
				Zagreb	66	1.788	0.412	Split	Zagreb	-0.163	0.105	-1.556	0.269
**I.12. Ever contacted ethical committee**	1.529	5.633	0.005	Rijeka	22	1.591	0.503	Rijeka	Split	-0.329	0.108	-3.056	0.008
				Split	25	1.920	0.277		Zagreb	-0.273	0.091	-3.007	0.009
				Zagreb	66	1.864	0.346	Split	Zagreb	0.056	0.087	0.651	0.792
**II.1.1. Experiencing DNACPR Decisions**	5.032	6.132	0.003	Rijeka	21	2.048	0.805	Rijeka	Split	0.684	0.195	3.500	0.002
				Split	22	1.364	0.581		Zagreb	0.364	0.162	2.243	0.069
				Zagreb	60	1.683	0.596	Split	Zagreb	-0.320	0.160	-2.003	0.117
**II.1.2. Experiencing withholding LST**	2.896	3.621	0.030	Rijeka	21	2.000	0.775	Rijeka	Split	0.500	0.198	2.530	0.034
				Split	20	1.500	0.607		Zagreb	0.362	0.161	2.248	0.068
				Zagreb	58	1.638	0.583	Split	Zagreb	-0.138	0.164	-0.841	0.679
**II.1.3. Experiencing withdrawing of artificial ventilation**	1.197	1.575	0.212										
**II.1.4. Experiencing withdrawing of endotracheal tube**	0.655	1.031	0.360										
**II.1.5. Experiencing withdrawing of inotropes**	0.027	0.029	0.972										
**II.1.6. Experiencing withdrawing of antibiotics**	2.521	3.238	0.043	Rijeka	21	1.476	0.602	Rijeka	Split	-0.251	0.190	-1.319	0.388
				Split	22	1.727	0.827		Zagreb	0.143	0.158	0.903	0.640
				Zagreb	60	1.333	0.542	Split	Zagreb	0.394	0.156	2.533	0.034
**II.1.7. Experiencing withdrawing of hydration**	0.582	2.786	0.066										
**II.2. Recording of DNACPR Decisions**	7.379	5.115	0.008	Rijeka	22	2.500	0.802	Rijeka	Split	0.750	0.251	2.992	0.010
				Split	24	1.750	0.897		Zagreb	0.576	0.209	2.754	0.019
				Zagreb	66	1.924	0.847	Split	Zagreb	-0.174	0.202	-0.861	0.666
**II.3. Compliance with DNACPR Decisions**	6.102	6.087	0.003	Rijeka	22	2.955	0.213	Rijeka	Split	22	2.955	0.213	0.045
				Split	21	2.238	0.944		Zagreb	21	2.238	0.944	0.206
				Zagreb	66	2.455	0.727	Split	Zagreb	66	2.455	0.727	0.089
**II.4. Recording of LST Limitation Decisions**	0.383	0.294	0.746										
**II.5.1. Involvement in LST limitation decision-making**	0.161	0.458	0.634										

**II.6. Initiator of LST limitation discussions**	3.965 1.655 0.197		
**II.7.2. LST limitation decision-making process includes physicians (ICU and others involved in treatment)**	0.017 0.005 0.995		
**II.7.3. LST limitation decision-making process includes physicians and nurses**	3.596 0.986 0.377		
**II.7.4. LST limitation decision-making process includes consultation of ethics committee**	1.758 0.482 0.619		
**II.8. Family members/legal guardians involvement in LST limitation decision-making**	2.192 1.361 0.261		
**II.11. Frequency of No Consensus Among Physicians**	2.724 7.559 < .001	Rijeka 21 2.048 0.384<br>Split 23 1.696 0.470<br>Zagreb 66 2.091 0.420	Rijeka Split 0.352 0.128 2.747 0.019<br>Zagreb -0.043 0.106 -0.407 0.913<br>Split Zagreb -0.395 0.103 -3.846 < .001
**II.12. Frequency of No Consensus among Physicians and Family members**	0.189 0.338 0.714		
**II.13. Disagreement with LST limitation decisions**	0.854 1.167 0.315		
**II.15. Time from Decision to Treatment Withdrawal**	3.994 2.140 0.123		
**III.4. Ethical acceptability of LST limitation**	4.380 6.530 0.002	Rijeka 22 2.682 0.568<br>Split 25 2.600 0.500<br>Zagreb 66 2.242 0.609	Rijeka Split 0.082 0.169 0.483 0.879<br>Zagreb 0.439 0.143 3.082 0.007<br>Split Zagreb 0.358 0.136 2.629 0.026
**III.5. Ethical difference between withholding and withdrawing**	2.812 2.200 0.116		
**III.6. Ethical acceptability of DNACPR**	5.945 7.380 < .001	Rijeka 22 2.727 0.456<br>Split 25 2.800 0.408<br>Zagreb 66 2.303 0.744	Rijeka Split -0.073 0.186 -0.392 0.919<br>Zagreb 0.424 0.156 2.715 0.021<br>Split Zagreb 0.497 0.149 3.335 0.003
**III.7. Ethical acceptability of limitation of hydration alongside limitation of LST**	1.970 4.023 0.021	Rijeka 22 1.045 0.213<br>Split 25 1.440 0.821<br>Zagreb 66 1.182 0.389	Rijeka Split -0.395 0.145 -2.728 0.020<br>Zagreb -0.136 0.122 -1.119 0.504<br>Split Zagreb 0.258 0.116 2.222 0.072
**III.8. Ethical equality of different end-of-life cases (brain dead, terminal, and patients in [irreversible] vegetative state)**	0.074 0.060 0.941		
**III.10. Ethical equality of LST limitation decision-making involving adult and pediatric patients**	2.457 2.844 0.062		
**III.13. Competent patient's expressed (verbally or written) decisions should be respected**	0.088 0.212 0.809		

**III.16. Frequency of being informed about patient or family wishes regarding LST limitation**	8.480 3.017 0.053		
**III.17. Usefulness of ACD in LST limitation decision-making**	1.332 1.066 0.348		
**III.18. Frequency of encountering ACD**	1.183 2.937 0.057		
**III.19. Existence of (your) individual legal responsibility regarding LST limitation decisions and their implementation**	5.564 4.306 0.016	Rijeka 22 2.273 0.767<br>Split 25 2.440 0.768<br>Zagreb 63 1.921 0.829	Rijeka Split -0.167 0.235 -0.712 0.757<br>Zagreb 0.352 0.199 1.769 0.185<br>Split Zagreb 0.519 0.190 2.734 0.020
**III.9.1. Good Medical Practice**	1.260 0.836 0.436		
**III.9.2. Patient's Interest**	1.440 1.697 0.188		
**III.9.3. Patient's Autonomy**	2.957 2.181 0.118		
**III.9.4. Treatment Costs**	25.571 8.558 < .001	Rijeka 22 1.773 0.813<br>Split 25 2.800 1.323<br>Zagreb 65 3.015 1.293	Rijeka Split -1.027 0.357 -2.875 0.013<br>Zagreb -1.243 0.301 -4.122 < .001<br>Split Zagreb -0.215 0.288 -0.749 0.735
**III.9.5. ACDs**	0.170 0.140 0.870		
**III.9.6. Wishes of Family/Legal Representatives**	6.180 4.748 0.011	Rijeka 22 3.227 0.869<br>Split 25 3.760 0.926<br>Zagreb 66 3.833 0.736	Rijeka Split -0.533 0.236 -2.259 0.066<br>Zagreb -0.606 0.199 -3.052 0.008<br>Split Zagreb -0.073 0.189 -0.387 0.921
**III.9.7. Legal regulations**	8.755 5.122 0.007	Rijeka 22 4.409 0.959<br>Split 25 4.000 0.957<br>Zagreb 65 3.692 0.900	Rijeka Split 0.409 0.270 1.514 0.288<br>Zagreb 0.717 0.228 3.143 0.006<br>Split Zagreb 0.308 0.218 1.414 0.337
**III.9.8. Religious Principles**	10.653 4.580 0.012	Rijeka 22 2.682 0.945<br>Split 25 3.080 1.115<br>Zagreb 65 3.462 1.105	Rijeka Split -0.398 0.315 -1.263 0.419<br>Zagreb -0.780 0.266 -2.931 0.011<br>Split Zagreb -0.382 0.254 -1.503 0.293
**III.9.9. Need for Vacant ICU Beds**	15.033 6.717 0.002	Rijeka 22 1.000 0.000<br>Split 25 1.800 1.080<br>Zagreb 64 1.953 1.214	Rijeka Split -0.800 0.309 -2.587 0.029<br>Zagreb -0.953 0.261 -3.646 0.001<br>Split Zagreb -0.153 0.249 -0.614 0.813
**III.11. Resource allocation considerations**	10.207 4.208 0.017	Rijeka 22 1.773 1.020<br>Split 25 2.320 1.249	Rijeka Split -0.547 0.322 -1.700 0.210<br>Zagreb -0.792 0.273 -2.897 0.013

				Zagreb	62	2.565	1.065	Split	Zagreb	-0.245	0.261	-0.937	0.618
**III.14. Different religious/cultural beliefs of patient or legal representative**	9.914	8.765	< .001	Rijeka	22	3.227	1.066	Rijeka	Split	0.027	0.220	0.124	0.992
				Split	25	3.200	0.764		Zagreb	-0.588	0.185	-3.170	0.006
				Zagreb	65	3.815	0.610	Split	Zagreb	-0.615	0.177	-3.477	0.002
**III.15. Different religious/cultural beliefs of physician**	15.000	6.567	0.002	Rijeka	22	1.682	0.894	Rijeka	Split	-0.358	0.312	-1.147	0.488
				Split	25	2.040	0.935		Zagreb	-0.896	0.264	-3.394	0.003
				Zagreb	64	2.578	1.166	Split	Zagreb	-0.538	0.252	-2.135	0.088
