# Supplementary material for: Navigating the shadows: medical professionals’ values and perspectives on end-of-life care within pediatric intensive care units in Croatia
Source: Front Pediatr. 2024 Aug 12;12:1394071. doi: 10.3389/fped.2024.1394071 (PMC11345198; doi:10.3389/fped.2024.1394071)
Supplement: Supplementary file 2 [file Table2.pdf]

Supplementary Table 2. Experiences, attitudes and value of Croatian pediatric ICU professionals regarding LST limitation – analysis by work experience

			Spearman rho		p
I.6. Experience	-	I.11. Awareness of Contact for Ethical Dilemma	-0.239	*	0.012
I.6. Experience	-	I.12. Contacted Ethical Committee	-0.133		0.162
I.6. Experience	-	II.1.1. Decision for CPR Non-resuscitation	-0.085		0.394
I.6. Experience	-	II.1.2. Withholding Therapeutic Procedures	0.044		0.664
I.6. Experience	-	II.1.3. Withdrawing Artificial Ventilation	0.001		0.990
I.6. Experience	-	II.1.4. Removing Endotracheal Tube	-0.039		0.698
I.6. Experience	-	II.1.5. Use of Inotropes	0.177		0.077
I.6. Experience	-	II.1.6. Use of Antibiotics	0.122		0.221
I.6. Experience	-	II.1.7. Decision on Hydration	0.053		0.600
I.6. Experience	-	II.2. Recording Decisions on CPR	-0.147		0.123
I.6. Experience	-	II.3. Compliance with DNR Orders	-0.279	*	0.003
I.6. Experience	-	II.4. Implementing Instructions for LST	-0.109		0.255
I.6. Experience	-	II.5.1. Involvement in End-of-Life Decisions	0.113		0.247
I.6. Experience	-	II.6. Initiator of End-of-Life Discussion	-0.084		0.448
I.6. Experience	-	II.7.2. Physician Council Decision Making	-0.095		0.337
I.6. Experience	-	II.7.3. Physicians and Nurses Decision-Making	-0.120		0.241
I.6. Experience	-	II.7.4. Consulting Ethical Committee	0.147		0.146
I.6. Experience	-	II.8. Family Involvement in Decision Making	-0.115		0.229
I.6. Experience	-	II.11. Frequency of No Consensus Among Physicians	0.073		0.452
I.6. Experience	-	II.12. Frequency of No Consensus of Physicians with Family	-0.081		0.402
I.6. Experience	-	II.13. Disagreement with Limiting Procedures	-0.016		0.866
I.6. Experience	-	II.15. Time from Decision to Treatment Withdrawal	0.055		0.581
I.6. Experience	-	III.4. Ethical Acceptability of Limiting Life-Sustaining Procedures	-0.050		0.600
I.6. Experience Doctor/Nurse	-	III.5. Ethical Difference Between Withholding and Withdrawing	-0.037		0.697
I.6. Experience	-	III.6. Ethical Acceptability of Non-Resuscitation	-0.051		0.591
I.6. Experience	-	III.7. Agreement on Stopping Hydration	0.057		0.551
I.6. Experience	-	III.8. Equality of Decision Making in Different Patient States	-0.108		0.257
I.6. Experience	-	III.9.1. Good Medical Practice	-0.099		0.300
I.6. Experience	-	III.9.2. Patient's Interest	0.047		0.624
I.6. Experience	-	III.9.3. Patient Autonomy	-0.109		0.255
I.6. Experience	-	III.9.4. Treatment Costs	-0.064		0.505
I.6. Experience	-	III.9.5. Written Document Expressing Patient's Prior Wishes	-0.156		0.104
I.6. Experience	-	III.9.6. Wishes of Family/Legal Representatives	-0.117		0.221
I.6. Experience	-	III.9.7. Legal Provisions	0.188	*	0.048
I.6. Experience	-	III.9.8. Religious Principles	-0.092		0.339
I.6. Experience	-	III.9.9. Need for Vacant ICU Beds	0.032		0.741
I.6. Experience	-	III.10. Equality of Decision Making for Adult Patients	-0.187	*	0.048

Supplementary Table 2. Experiences, attitudes and value of Croatian pediatric ICU professionals regarding LST limitation – analysis by work experience

			Spearman	
			**rho**	**p**
I.6. Experience	-	III.11. Importance of Resource Allocation in End-of-Life Decisions	0.024	0.802
I.6. Experience	-	III.12. Decision Maker for Incapacitated Patients	-0.026	0.787
I.6. Experience	-	III.13. Respect for Patient's Expressed Decision	-0.143	0.133
I.6. Experience	-	III.14. Respect for Different Religious/Cultural Beliefs Expressed by Patient or Representative	-0.231 *	0.014
I.6. Experience	-	III.15. Respect for Different Religious/Cultural Beliefs Expressed by Physician	4.024×10^{-4}	0.997
I.6. Experience	-	III.16. Frequency of Being Informed About Patient or Family Wishes	0.007	0.941
I.6. Experience	-	III.17. Usefulness of Written Patient Wishes in Decision Making	-0.273 *	0.004
I.6. Experience	-	III.18. Frequency of Encountering Written Patient Wishes	0.006	0.948
I.6. Experience	-	III.19. Personal Legal Responsibility in Decision Making	0.006	0.954

* p < .05
