# Supplementary material for: Navigating the shadows: medical professionals’ values and perspectives on end-of-life care within pediatric intensive care units in Croatia
Source: Front Pediatr. 2024 Aug 12;12:1394071. doi: 10.3389/fped.2024.1394071 (PMC11345198; doi:10.3389/fped.2024.1394071)
Supplement: Supplementary file 3 [file Datasheet1.pdf]

»ETHICAL ATTITUDES UNDERLYING DECISIONS ON LIFE-SUSTAINING TREATMENT IN PATIENTS AT THE END-OF-LIFE IN INTENSIVE CARE UNITS (ICU)«

I. DATA

1. Hospital:
   1. UCH SISTERS OF MERCY
   2. UCH ZAGREB PETROVA
   3. UCH HOLY SPIRIT
   4. UCH ZAGREB
   5. UCH SPLIT
   6. UCH RIJEKA

2. Age (in years): ________________

3. Gender (circle): 1. M

   2. F

4. Status:
   1. Physician specialist
   2. Nurse-college graduate
   3. Nurse-high school graduate
   4. Nurse-university graduate

5. How many years you work as physicians/nurse: ______________________
6. How many years you work you work in ICU: ___________________________
7. Your medical specialization (for physicians): ___________________________

8. Type of work in ICU:
   1. Regular – I work every day in ICU
   2. Occasional rad u JIM-u – I work in ICU only “on duty” or due to lack of staff

9. Do you know who is running ethical committee at your hospital?
   1. Yes
   2. No

10. Do you know whom to reach when encountering ethical dilemmas?
    1. Yes
    2. No

11. Have you ever contacted the ethical committee in your institution?
    1. Yes
    2. No

II. EXPERIENTIAL – CONDUCT PART

In the following questions, we are interested in your involvement and participation in limiting life-sustaining procedures for patients at the end of life.

1.) What method of limiting therapeutic life-sustaining procedures do you choose in your ICU? Are the following statements valid? (circle)

1.1. Not-attempting cardiopulmonary reanimation
3. Frequently
2. Rarely
1. Never

1.2. Withholding of therapeutic procedures– eng. *withholding*
3. Frequently
2. Rarely
1. Never

Withdrawing therapeutic procedures – eng. *withdrawing*:

1.3. Artificial ventilation
3. Frequently
2. Rarely
1. Never

1.4. Removing of endotracheal tube
3. Frequently
2. Rarely
1. Never

1.5. Inotropes
3. Frequently
2. Rarely
1. Never

1.6. Antibiotics
3. Frequently
2. Rarely
1. Never

1.7. Hidration
3. Frequently
2. Rarely
1. Never

1.8. Other (describe): ________________________________

2.) Are decisions not to apply cardiopulmonary resuscitation in patients at the end-of-life made and recorded in your ICU?

3. Yes, in writing
2. Yes, verbally
1. No

3.) Do you respect the instruction not to apply cardiopulmonary resuscitation methods?

3. Always
2. Rarely
1. Never

4.) Do you use verbal or written instructions to limit life-sustaining procedures?

3. Yes, oral
2. Yes, written*
1. No such instructions are used

*if YES: Do you have special form? (you can attach it to the questionnaire)

2. Yes

1.No

5.) Were you involved in the decision-making process regarding limitation of life-sustaining procedures for patients at the end-of-life?

5.1. 2. Yes

5.2. How many times during this year ________________

5.3. How many times during last five years ____________

What was the leading reason:

5.4. Brain death

5.5. Terminal disease

5.6. Vegetative state

5.7. Other ____________________________

1. No

6.) Who started the conversation about limitation of life-sustaining procedures

1. On physician's initiative
2. On nurse's initiative
3. On family members or legal representatives' initiative
4. 1+2
5. 1+3

7.) How does decision-making in your ICU go (circle)?

(1= strongly agree; 2 = agree; 3 = cannot decide; 4 = disagree; 5 = strongly disagree)

7.1. I decide alone	5	4	3	2	1
7.2. Decision-making includes physicians	5	4	3	2	1
7.3. Decision-making includes physicians and nurses	5	4	3	2	1
7.4. I ask hospitals ethical committee	5	4	3	2	1

8.) Is the following statement valid: "Family members/legal representatives are also involved in decision-making?"

4. Always

3.Usually

2.Rarely

1.Never

9.) What is the further decision-making procedure, if no agreement was reached between the physicians? (describe)

___

10.) What is the further decision-making procedure, if no agreement was reached between physicians and family members/legal representatives? (describe)

___

11.) How often is consensus not reached among physicians?

3. Frequently
2. Rarely
1. Never

12.) How often is consensus not reached among physicians and family members/legal representatives?

3. Frequently
2. Rarely
1. Never

13.) Have you ever disagreed with a mode of limitation of life-support procedures?

3. Frequently
2. Rarely
1. Never

14.) Have you ever refused to participate in the decision-making process regarding the limitation of life-support procedures or their implementation?

Yes*

3. Frequently
2. Rarely
1. Never

*If YES, describe why:

___

15. How long does it take from the decision to the limitation of life-support of procedures?

5. Immediately
4. Immediately after decision is made and family agreed
3. Around 6 hours
2. Around 24 hours
1. More than 24 hours

- If more than 24 hours: What was the reason for such a delay? (describe):

___

III. ATTITUDES

In the following questions, we are interested in your attitudes towards limitation of life-sustaining procedures for patients at the end of life.
There are no right or wrong answers.

1.) What does the term "patient at the end of life" mean for you:

1. A patient who is expected to die within the next 12 months, which includes patients whose death is inevitable within the next few hours or days, due to worsening of the underlying disease or sudden deterioration of the health
2. A patient who is expected to die in the next few hours or days, due to the worsening of the underlying disease or a sudden worsening of the health
3. Other (describe): ______________________________

2.) What, in your opinion, defines the term "withholding from the introduction of therapeutic procedures" - eng. withholding?

1. No other new drugs or other treatment methods are introduced, all other methods which patient is already receiving are kept
2. Medicines that do not improve the patient's condition are discontinued
3. Patient is not being reanimated by cardiopulmonary resuscitation, but all other therapeutic procedures are continued
4. All medications except fluids are discontinued
5. Other (describe): __________
6. 1+4
7. 1+2+4
8. 1+2
9. 2+3
10. 1+3
11. 1+2+3

3.) What, in your opinion, defines the term „withdrawing of therapeutic procedures" – eng. *withdrawing*?

1. All drugs are withdrawn expect analgesics and sedatives
2. All drugs are withdrawn, and patient is disconnected form respiration if being on one
3. All drugs are withdrawn, and patient is disconnected form respiration if being on one, and endotracheal tubes is removed
4. All drugs are withdrawn, and supportive treatment is stopped (dialysis, extracorporeal blood flow, etc.)
5. Other (describe): ____________________
6. 1+2
7. 1+4
8.

4.) Are, in your opinion, decisions to limit life-sustaining procedures in the sense of withholding and withdrawing of therapeutic procedures in patients at the end-of-life ethically acceptable?

3. Yes
2. No
1. Cannot decide

5.) For you, is there significant difference in ethical sense between withholding and withdrawing of therapeutic procedures?

3. Yes
2. No
1. Cannot decide

6.) Are, in your opinion, decisions not to reanimate by cardiopulmonary resuscitation in patients at the end-of-life ethically acceptable?

3. Yes
2. No
1. Cannot decide

7.) Do you agree that when limiting life-sustaining treatments for patients at the end-of-life, hydration should also be discontinued?

3. Yes*
2. No
1. Cannot decide

7.1 If YES: do you agree even if that could be expected direct cause of the patient's death at the end-of-life?

3. Yes
2. No
1. Cannot decide

8.) Is, in your opinion, decision-making on limitation of life-sustaining treatments ethically equal in the following cases: in a patient who is brain dead or terminally ill or in an (irreversible) vegetative state?

3. Yes
2. No*
1. Cannot decide

8.1 If NO, in which cases decisions are not the same and why?
(describe):____

9.) To what extent do you agree with the following statement: "When deciding on limiting life support treatments, the following aspects must be respected":
(Please circle the answer that applies to you).

(1= strongly agree; 2 = agree; 3 = cannot decide; 4 = disagree; 5 = strongly disagree)

9.1 . Good medical practice	5	4	3	2	1
9.2. Patient’s interest	5	4	3	2	1
9.3. Patient’s autonomy	5	4	3	2	1
9.4. Treatment costs	5	4	3	2	1
9.5. Advance care directives	5	4	3	2	1
9.6. Wishes of Family/Legal Representatives	5	4	3	2	1
9.7. Legal regulations	5	4	3	2	1
9.8. Religious principles	5	4	3	2	1
9.9. Need for vacant ICU beds	5	4	3	2	1

10.) For you, is the decision to limit life-sustaining treatments ethically equal if the patient in the above question is an adult?

3.Yes
2.No*
1. Cannot decide

10.1 If NO, in which cases decision is not equal to others and why (describe):
____

11.) In your opination, are limited resource allocation considerations in healthcare important when deciding on the further treatment of the patients at the end-if-life? (circle)

5. Very important
4. Important
3. Cannot decide
2. Not important
1. Not important at all

12.) Who should, in your opinion, decide on limitation of life-sustaining procedures if patient is incompetent/incapacitated?

1. Physician alone
2. Council of physicians
3. Physician + family/legal representatives
4. Hospitals ethics committee
5. Court
6. Legal representative based on advanced care directive
7. 2+4
8. 2+3+4
9. 3+4
10. 2+3
11. 2+3+6
12. 2+3+4+5+6
13. 2+3+4+6
14. 3+6
15. 2+6

13.) Do you think that patients' expressed decisions regarding limitation of life-sustaining treatments, verbal or written, should be respected it they are competent making those decisions?

3. Yes
1. No
2. Don't know

14.) To what do you agree with the following statement: „Different religious/cultural beliefs expressed by the patient or legal representative should be respected?"

5. Strongly agree
4. Agree
3. Cannot decide
2. Disagree
1. Strongly disagree

15.) To what do you agree with the following statement: Different religious/cultural beliefs expressed by the physicians should be respected?"

5. Strongly agree
4. Agree
3. Cannot decide
2. Disagree
1. Strongly disagree

16.) How often are you familiar with the wishes of the patient or his family (legal representatives) regarding the continuation of active treatment or the limitation of life-sustaining procedures for patients at the end-of-life?

5. Very often
4. Often
3. Cannot decide
2. Rarely
1. Very rarely

17.) Is written advanced care directive useful in reaching decisions regarding treatment of patients at the end-of-life?

5. Strongly agree
4. Agree
3. Cannot decide
2. Disagree
1. Strongly disagree

18.) How often do you encounter advance care directives in your practice?

3. Often
2. Rarely
1. Never

19.) Do you think that you are personally subject to legal/criminal liability when making and executing a decision to limit life-sustaining procedures?

3. Yes
1. No
2. Cannot decide
